# Selenite Protection of Tellurite Toxicity Toward *Escherichia coli*

**DOI:** 10.3389/fmolb.2015.00069

**Published:** 2015-12-18

**Authors:** Helen A. Vrionis, Siyuan Wang, Bronwyn Haslam, Raymond J. Turner

**Affiliations:** Department of Biological Sciences, University of CalgaryCalgary, AB, Canada

**Keywords:** metalloids, glutathione, tellurite, selenite, metal resistance

## Abstract

In this work the influence of selenite on metal resistance in *Escherichia coli* was examined. Both synergistic and antagonistic resistance and toxicities were found upon co exposure with selenite. In wild type cells co-exposure to selenite had little effect on arsenic resistance, decreased resistance to cadmium and mercury but led to a dramatically increased resistance to tellurite of 32-fold. Due to the potential importance of thiol chemistry in metal biochemistry, deletion strains in γ-glutamylcysteine synthetase (key step in glutathione biosynthesis, encoded by *gshA*), thioredoxin (*trxA*), glutaredoxin (*grxA*), glutathione oxidoreductase (*gor*), and the periplasmic glutathione transporter (*cydD*) were also evaluated for resistance to various metals in the presence of selenite. The protective effect of selenite on tellurite toxicity was seen in several of the mutants and was pronounced in the *gshA* mutant were resistance to tellurite was increased up to 1000-fold relative to growth in the absence of selenite. Thiol oxidation studies revealed a faster rate of loss of reduced thiol content in the cell with selenite than with tellurite, indicating differential thiol reactivity. Selenite addition resulted in reactive oxygen species (ROS) production equivalent to levels associated with H_2_O_2_ addition. Tellurite addition resulted in considerably lower ROS generation while vanadate and chromate treatment did not increase ROS production above that of background. This work shows increased resistance toward most oxyanions in mutants of thiol redox suggesting that metalloid reaction with thiol components such as glutathione actually enhances toxicity of some metalloids.

## Introduction

The increasing use of tellurite in industrial applications has resulted in release into the environment creating a health concern (Ding et al., 2002; Ba et al., 2010; Sandoval et al., 2010) and an interest in better understanding the molecular mechanism of tellurite toxicity. Toxicity of tellurite in many enteric bacteria occurs at concentrations as low as 1 μg/mL (Turner et al., 1999; Bajaj and Winter, 2014). A number of plasmid and chromosomally encoded determinants for tellurite resistance have been identified (Jobling and Ritchie, 1987; Walter and Taylor, 1989; Hill et al., 1993; Taylor et al., 1994; Turner et al., 1995a,b; Taylor, 1997) and though the mechanisms of resistance have not been fully elucidated in many of these, evidence has shown that the mechanism of resistance is not through increased efflux or reduced uptake of tellurite (Turner et al., 1995a).

Thiol redox enzymes (glutathione reductase and thioredoxin reductase) and their metabolites (thioredoxin, glutaredoxin, and glutathione) which are all part of the cellular thiol:redox buffering system have been shown to be involved in oxyanion chemistry (Turner et al., 1995b). Glutathione has been shown to be a key intermediate in cellular processing of selenium and is likely a primary target of tellurite reactivity (Turner et al., 2001). When species of microbes are exposed to tellurite one can observe a blackening of the media, which is the result of reduction of tellurite to elemental tellurium (Te^0^), which can accumulate as nanocrystals or nanoparticles (Turner et al., 2012). It has been proposed that glutathione is capable of mediating tellurite to elemental tellurium (Te^0^) reduction (Turner et al., 2001), and this reduction can be observed *in vitro*. The reaction may also be accompanied with generation of superoxide anions as has been seen with selenite (Bebien et al., 2002; Kessi and Hanselmann, 2004).

In response to environmental stresses, microbes can exhibit a variety of protective mechanisms. Pre-exposure to a contaminant at sub lethal levels can lead to increased resistance to subsequent exposure of the same stressor (adaptive response) or exposure to a different stress (cross-protective response; Vattanaviboon et al., 2003). Such protective effects have been observed to contribute to adaptation to oxidative stress and metal toxicity (Banjerdkij et al., 2005). In *Xanthomonas campestris* for instance, cadmium exposure has been shown to provide cells with protection against peroxide killing (Banjerdkij et al., 2005). In the present work, co-exposure of *Escherichia coli* strains to selenite is seen to increase resistance to a number of metals including tellurite. Being in the same group in the periodic table does not guarantee equivalent ROS production and this was observed in this work where the ROS production by tellurite was considerably lower than that of selenite. This was in line with previous observations (Tremaroli et al., 2007) suggesting that the protective effect provided by selenite may be related to it's triggering a stronger adaptation to oxidative stress.

## Materials and methods

### Strains and growth conditions

Strains utilized in this work was derived from the Keio collection (Datsenko and Wanner, 2000; Baba et al., 2006). Strain *K12* (W3110) BW25113 *(F (araD-araB)567_lacZ4787(::rrnB-3) _rph-1 _(rhaD-rhaB) 568 hsdR514* was used as Wild type. Subsequent mutants contain a Kanamycin insertion into the designated gene: JW2663 (Δ*gshA)*, JW3467 *(*Δ*gor)*, JW0833 *(*Δ*grxA)*, JW5856 *(*Δ*trxA), and* JW0870 *(*Δ*cydD).* Cultures were streaked from frozen stocks onto LB media with 40 μg/ml Kan and grown at 37°C for 24 h—these first streaks (maintenance cultures-MC) were stored for approximately 2 weeks at 4°C. As needed, fresh second streaks or liquid cultures were made from these cultures and grown for 24 h (over-night) prior to experimentation. Under the culture conditions used in this work there was no significant difference in growth rate observed for the mutants relative to WT.

### Stock solutions

Metal salts were obtained from Sigma Chemical Company (St. Louis, U.S.A.) for the various metal(loids): selenite (Na_2_SeO_3_), tellurite (Na_2_TeO_3_), vanadate (NaVO_3_), chromate (Na_2_CrO_4_), arsenite (NaAsO_2_), cadmium (CdCl_2_), and mercury (HgCl_2_) were diluted in sterile water at twice the highest concentration to be tested (Turner et al., 2001). Metal solutions were passed through a 0.22 μm syringe into sterile vials and stored at room temperature.

### Minimal inhibitory concentration (MIC)

Minimal inhibitory concentration was assessed in 96 well microtitre plates. One column served as sterile control and one as a no metal control. Ten columns were used for susceptibility testing for the log-2 dilution series of the metal(loid). Two rows for each metal were used for technical replicates within a plate. Each experiment was performed three times thus providing 6 replicates for each metal concentration. Metal concentration ranges tested were 4096–8 μg/ml arsenite, 410–0.4 μg/ml chromate, 3200–31.3 μg/ml vanadate, 40000–390 μg/ml selenite, and 512–0.025 μg/ml tellurite. A working solution of metal was prepared (highest concentration to be tested) in Luria Bertaini broth media and subsequent concentrations achieved by serial dilution. Inoculums were prepared as previously described (Harrison et al., 2005). Briefly, a second sub-culture was prepared from MC 24 h prior to susceptibility testing experiments and used to create a standard matching a 1.0 McFarland Standard. This solution was diluted 1/30 in LB and 10 μL of this dilution was added to each well of the microtitre plate. Total volume in each well was 210 μl (200 μl LB and 10 μl inoculum).

An adaptation of the interaction index defined by Berenbaum can be defined by ΣFIC = FIC_A_ + FIC_B_ = MIC_A_/MIC_AB_ + MIC_B_/MIC_BA_; where MIC_A_, MIC_B_ are the MICs of metals A and B acting alone and MIC_AB_, MIC_BA_ are the MICs of metals acting in combination. ΣFIC_min_ is the lowest ΣFIC when synergy is occuring or the highest, ΣFIC_max_, for antagonism (Berenbaum, 1977; Bellio et al., 2015). In this approach an FIC value of less than 0.5 indicates synergy while values above 4 indicate antagonism.

### Thiol assays

Over-night cultures were diluted 1/100 in M9 media and grown at 37°C (250 rpm) to mid-log phase (O.D_0.600_ ~ 0.5). This bulk culture was then divided into four 25 mL aliquots in sterile flasks containing a stir-bar and moved to a stir plate placed at room temperature. The four test conditions settled on for this work were as follows: control (no metal added); tellurite (1 μg/mL); selenite (800 μg/mL) and tellurite + selenite (1 μg/mL and 800 μg/mL respectively). After an ~5 min room temperature equilibration, time zero samples were collected for determination of initial protein and thiol content. 1 mL samples were collected and pelleted by centrifugation and the supernatant discarded. Samples were then immediately frozen in liquid N_2_ while the pellets for protein determination were washed once with water prior to flash-freezing. Samples were stored at −80°C until analysis. Subsequent samples were taken over the 2 h exposure. Samples for protein concentration determination were taken at the 0, 1, and 2 h time points.

Thiol determination was by the dithiol-nitrobenzoate (DTNB) assay (Turner et al., 1999). In brief, a solution containing 0.1 mm DTNB, 5 mM EDTA, 50 mM Tris pH = 8 and 0.1% SDS was prepared. The lysis was performed in the presence of DTNB so that reaction with DTNB would be rapid. It is possible that some thiol oxidation would occur during the cell lysis but this is expected to be constant across all samples. Each sample pellet after thawing on ice was resuspended by vortexing with 1 mL of DTNB solution and incubated at 37°C for 45 min. Samples were pelleted by centrifugation and the Absorbance of the supernatants at 412 nm was measured. An extinction coefficient for DTNB of 1.36 × 10^4^ M^−1^ cm^−1^ was used to determine reduced thiol concentration. Protein concentration was determined using the Lowry method (Lowry et al., 1951) with bovine serum albumin (BSA) used to develop a standard curve.

### Reactive oxygen species (ROS) assay

An overnight culture was diluted 1/100 in fresh LB and grown at 37°C (250 rpm) to mid-log phase (O.D_0.600_ ~ 0.5). Cells were pelleted, washed one time in 0.9% saline and then resuspended in an equivalent volume of 0.9% saline. A small aliquot was collected and pelleted for protein determination. DCFA was added to the cell/saline solution from a 2 mM Stock to a final concentration of 5 μM. DCFA is light sensitive so from here on all solution flasks/vials were kept covered. The cell/saline/dye solution (sample) was shaken for 1 h at room temperature. The sample solution was then distributed in 4 mL aliquots into cuvettes and appropriate volumes of metals were added from metal stock solutions to obtain desired final concentrations (128 μg/ml ${\text{AsO}}_{2}^{-}$, 512 μg/ml ${\text{AsO}}_{2}^{-}$, 13 μg/ml Cr_2_O_7_, 16384 μg/ml Se_2_O_4_, 800 μg/ml Se_2_O_3_, 0.125, 0.25, 1, and 25 μg/ml TeO_3_, 800 μg/ml VO_3_). Cell/metal mixes were quickly mixed by inversion and time marked as *t* = 0. No metal and 1% H_2_O_2_ addition were used as negative and positive controls respectively.

### Protein isolation

Over-night cultures were diluted 1/100 in multiple 3 L flasks containing 1 L of fresh LB and culture grown to an O.D_0.600_ ~ 0.45–0.5. Cells were harvested by centrifugation at 6000 rpm for 10 min. at 4°C. Cells were then washed with 25 mM K_2_HPO_4_/75 mM NaCl (pH = 7) followed by another centrifugation. Cell pellets were thoroughly resuspended in 25 mM K_2_HPO_4_ with 2 mM Phenyl-methyl-sulfonyl fluoride (PMSF) and 0.03 mg/mL of DNAse I added. Cell suspensions (on ice) were then French pressed two times at 20,000 psi and subsequently centrifuged (10,000 rpm for 15 min) to remove unlysed cells and debris. Soluble and membrane fractions were separated by ultracentrifugation at 40,000 rpm for 1.5 h at 4°C. Samples were stored at −80°C until use.

### Superoxide dismutase (SOD) assay

Soluble cell fractions were obtained as described above with the following modifications. Pellets (from 100 mL of cells) were washed once in phosphate buffered saline (PBS) and subsequently resuspended in 2 mL of 50 mM Tris (pH = 7.8) treated with 100 μM PMSF. The cell suspensions were sonicated (on ice) on setting 50% power for two 1 min bursts with a 1 min pause in between and a final 10 s pulse. The suspensions were centrifuged at 7000 rpm @ 4°C for 15 min and the supernatants (crude extracts) subsequently ultracentrifuged at 35200 rpm for 1 h. The supernatants (soluble fractions) were collected and stored at −20°C.

Approximately 35 μg of soluble fraction for each of the samples were electrophoresed in non-denaturing 8% polyacrylamide gels at 4°C and gel assay determination of SOD activity was performed by an *in situ* staining procedure as previously described (Beauchamp and Fridovich, 1971; Dhindsa et al., 1981; Borsetti et al., 2005). In brief, gels were equilibrated in 50 mM potassium phosphate buffer and then treated with a mixture of nitrotetrazolium blue, riboflavin, and TEMED in the dark. The solution was then poured away and gel(s) exposed to light. A dark band indicates SOD activity. Bands corresponding to SOD activity were analyzed by an Image Analyzer FLA-3000 (Fujifilm, Japan).

## Results

### Susceptibility to metals and co-exposure

The susceptibility of *E. coli* wild type and mutants of thiol/redox homeostasis (Δ*gshA*, Δ*grxA*, Δ*trxA*, Δ*gor, and* Δ*cydD*) toward NaAsO_2_, CdCl_2_, HgCl_2_, Na_2_TeO_3_, and Na_2_SeO_3_ was assayed to determine their MIC. The nature of the two-log dilution, differences in MIC at the low concentration range are less meaningful than changes at higher concentrations. All strains exhibited a high inherent resistance to NaAsO_2_ and CdCl_2_ (256-1024 μg/mL and 64-256 μg/mL respectively), while being less resistant to HgCl_2_ (~2 μg/mL) and ${\text{TeO}}_{3}^{2-}$ (~0.25 μg/mL) (Table 1).

**Table 1:** **Evaluation of synergistic and antagonistic responses of the addition of selenite on the minimal inhibitory concentration (MIC) toward other metal(loid)s**.

		**Selenite addition μg/mL**						
		**0**	**100**	**200**	**400**	**800**	**1600**	**3200**
**Strain**	**Metal(loid)**	**MIC (μg /mL)**						
WT	${\text{AsO}}_{2}^{-}$	512	512	512	512	512	256	< 8
	Cd^2+^	256	128	16	< 8	< 8	< 8	< 8
	Hg^2+^	2	1	1	1	< 0.5	< 0.5	< 0.5
	${\text{TeO}}_{3}^{2-}$	1	2	4	8	16	32	2
ΔtrxA	${\text{AsO}}_{2}^{-}$	256	256	256	128	128	< 8	< 8
	Cd^2+^	256	64	16	< 8	< 8	< 8	< 4
	Hg^2+^	2	< 0.5	< 0.5	< 0.5	< 0.5	< 0.5	< 0.5
	${\text{TeO}}_{3}^{2-}$	0.25	4	16	64	128	32	16
ΔgrxA	${\text{AsO}}_{2}^{-}$	512	256	256	128	128	16	< 8
	Cd^2+^	256	64	16	< 8	< 8	< 8	< 8
	Hg^2+^	2	2	1	1	0.5	1	< 0.5
	${\text{TeO}}_{3}^{2-}$	0.25	2	4	32	32	32	< 1
ΔgshA	${\text{AsO}}_{2}^{-}$	512	256	256	128	128	16	< 8
	Cd^2+^	128	64	32	32	< 8	< 8	< 8
	Hg^2+^	2	2	< 0.5	< 0.5	< 0.5	< 0.5	< 0.5
	${\text{TeO}}_{3}^{2-}$	0.25	64	128	256	256	64	< 1
Δgor	${\text{AsO}}_{2}^{-}$	512	512	512	256	32	8	< 8
	Cd^2+^	64	64	32	8	< 8	< 8	< 8
	Hg^2+^	2	1	< 0.5	< 0.5	< 0.5	< 0.5	< 0.5
	${\text{TeO}}_{3}^{2-}$	0.25	4	16	32	64	32	< 1
ΔcydD	${\text{AsO}}_{2}^{-}$	1024	1024	1024	512	512	64	< 8
	Cd^2+^	128	64	32	< 8	< 8	< 8	< 8
	Hg^2+^	2	1	1	< 0.5	< 0.5	< 0.5	< 0.5
	${\text{TeO}}_{3}^{2-}$	0.5	8	16	32	64	64	64

As expected from previous studies exploring tellurite, the MIC thiol redox mutants were less than wildtype (Turner et al., 1995b). The deletion of *gor* exhibited a slightly higher susceptibility to CdCl_2_ relative to the other strains. The Δ*trxA* mutant showed a loss of resistance to ${\text{AsO}}_{2}^{-}$. The Δ*cydD* mutant led to changes in MIC for ${\text{AsO}}_{2}^{-}$, Cd as well as ${\text{TeO}}_{3}^{2-}$.

The effect of cell co-exposure to the above metals with increasing (2-fold) concentrations of ${\text{SeO}}_{3}^{2-}$ (0-3200 μg/mL) was also examined (Table 1). The maximum of 3200 μg/ml was chosen, as this is the dilution before the MIC of Selenite. The goal was to see if selenite would protect cells from the mechanisms of other metal toxicities. This combined with the key thiol/redox homeostasis mutants would provide clues to biochemical processes. We considered that both synergistic and antagonistic results could be possible. Arsenite resistance was not affected by the presence of ${\text{SeO}}_{3}^{2-}$ until the selenite MIC was approached (~3200 μg/mL). CdCl_2_ resistance decreased very slowly at lower selenite concentrations (~0–200 μg/mL) but dropped to < 8 μg/ml when more than 400 μg/ml of ${\text{SeO}}_{3}^{2-}$ was present in the growth media. Cell resistance to HgCl_2_ did not change considerably with selenite co-exposure, while in contrast selenite provided remarkable protection against tellurite toxicity in all the strains, with the *gshA* mutant exhibiting up to 1000-fold higher MIC than wild type. It should be noted that the protective effect was specific to selenite (i.e., selenate did not provide protection, data not shown). The synergy occurring during co-treatment with tellurite and selenite was revealed by an interactive model where FIC values were below 0.5 indicating synergy; while FIC values for all other metals were above 4 reflecting the antagonism with these metals.

### Effect of ${\text{TeO}}_{3}^{2-}$, ${\text{SeO}}_{3}^{2-}$, and co-exposure on RSH content

**Figure 1:**
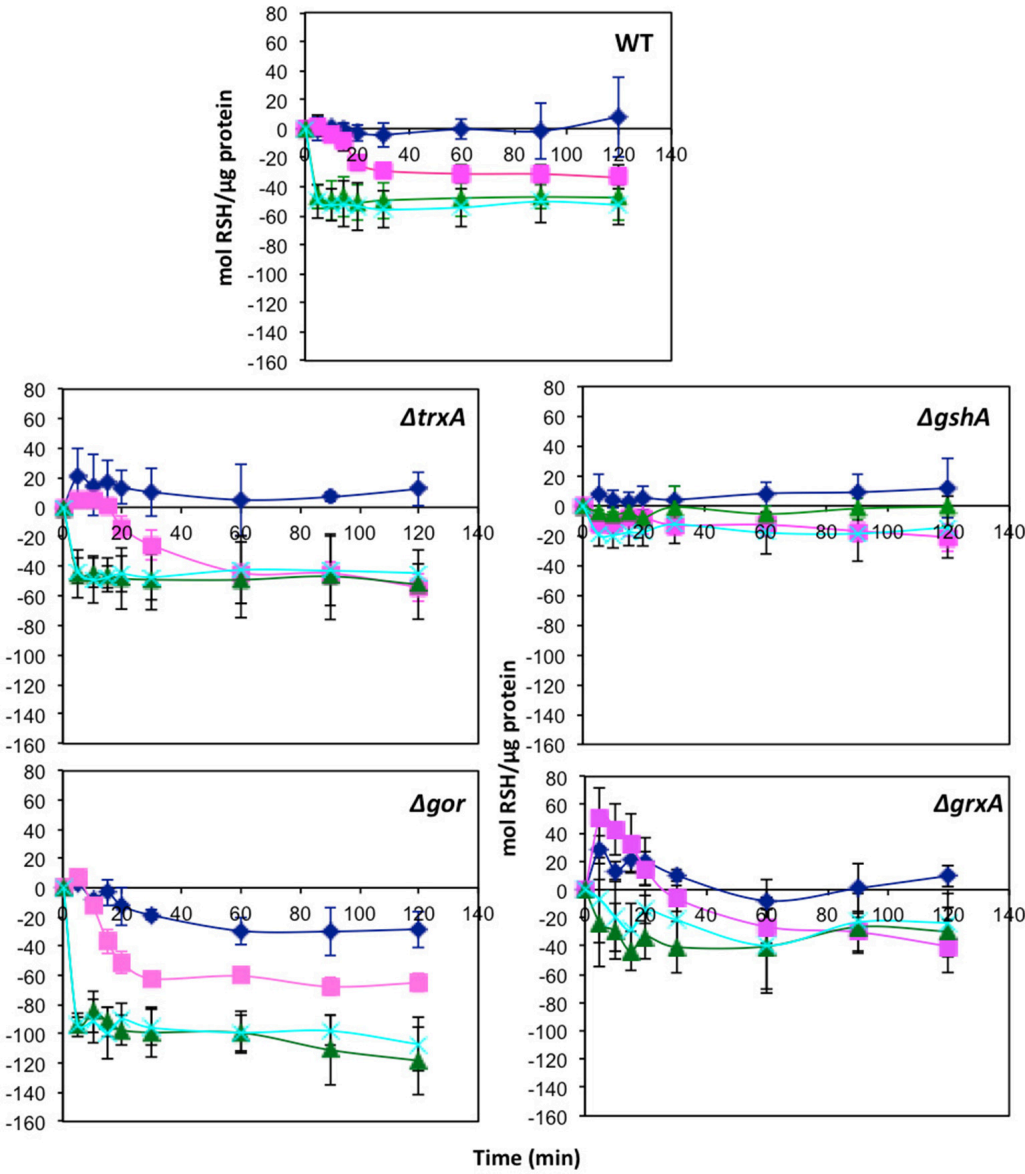
**Change in reduced thiol pool in response to various metalloid challenges**. Mid log phase cultures were exposed to metalloid challenges and their reduced thiol content was measured with time to evaluate the thiol oxidation rate. Control without challenge (blue diamonds), 1 μg/ml tellurite (red squares), 800 μg/ml selenite (green triangles), 1 μg/ml tellurite with 800 μg/ml selenite (cyan X). Wild type is in upper panel with each of four different redox balance mutants indicated. The error bars represent the standard deviation between 3 biological trials.

One of the targets of tellurite biochemistry is the reaction with free thiol groups mainly cysteine, which results in a depletion of cell RSH concentration (Turner et al., 2001). In order to examine if the mechanism of selenite protection is related to beneficial protection of the RSH pool, mid log phase cells were treated with either tellurite (1 μg/mL), selenite (800 μg/mL), or a mixture of the two and then changes in the RSH content over time monitored. Concentrations were chosen based on the MIC data in Table 1, where 800 μg/mL is the highest concentration with no inhibition found. Examination of the [RSH] with time curves in Figure 1 shows that in *E. coli* there is a rapid decrease in the RSH concentration in response to selenite addition. The response to tellurite is more gradual although the final RSH concentrations 2 h post-treatment are comparable. In the conditions when both Se and Te oxyanions are present, it is ${\text{SeO}}_{3}^{2-}$ biochemistry that dominates over tellurite in driving RSH reactions. Greater decreases in RSH concentration in the Δ*gor* mutant reflect the decreased ability of this strain to reduce oxidized GSSG. For the Δ*trxA* mutant [RSH] after 1 h was essentially identical in the tellurite and selenite (±tellurite) conditions. A similar trend was seen in the Δ*grxA* mutant. The Δ*gor* mutant exhibited the highest loss in [RSH] under all conditions including untreated. Furthermore, the Δ*gor* mutant exhibited the greatest difference in RSH content between the tellurite and selenite (±tellurite) samples. The low detection of RSH levels and the minimal change in RSH under all conditions in the Δ*gshA* mutant likely reflects the fact that the reactive (and DTNB detectable) [RSH] in *E. coli* is actually GSH.

### Production of reactive oxygen species in response to oxyanion exposure

Mid-log phase cells were washed and incubated with a membrane permeable dye carboxy-dichlorodihydrofluorescein diacetate (DCFA) (Mishra et al., 2005) that fluoresces upon reaction with a variety of reactive oxygen species (ROS) as a result in cleavage of acetate and ester groups. Fluorescence from control (untreated) cells was evaluated for 2 h post attack [ < 8000 arbitrary units (a.u.)] and background values were subtracted from subsequent fluorescence measurements.

**Figure 2:**
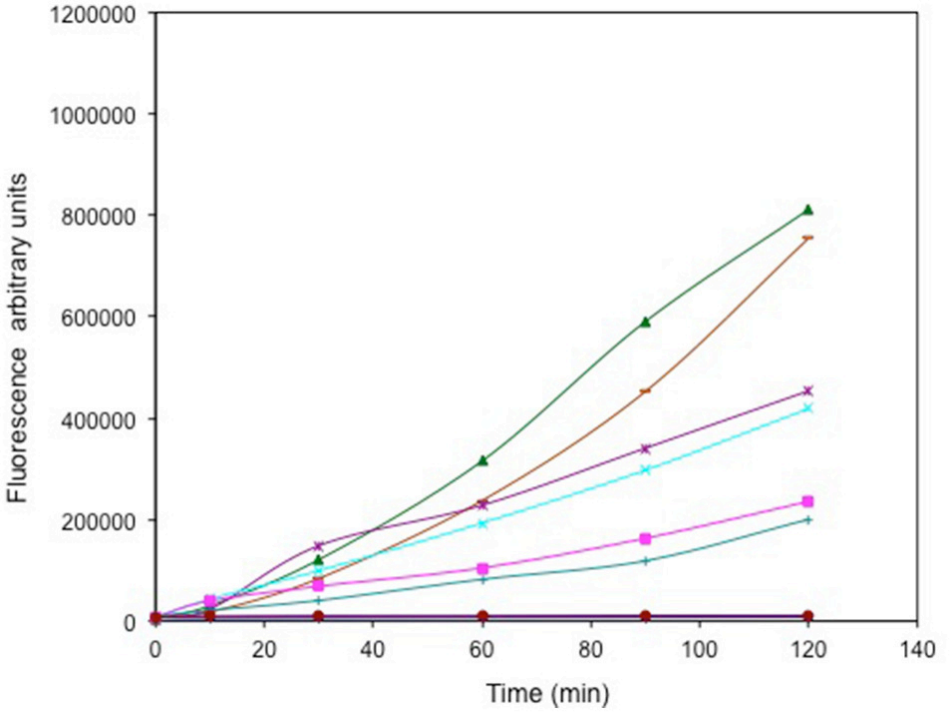
**Time course of reactive oxygen species (ROS) production**. Exponentially growing cells were washed, suspended in saline in the presence of the ROS sensitive probe carboxy-DCFH-DA and exposed to metals. 800 μg/mL selenite (green triangle), 16384 μg/mL selenate (purple ^*^), 512 μg/mL arsenite (cyan X), 1 μg/mL tellurite (pink square), 128 μg/mL arsenite (green +), 13 μg/mL chromate (blue line) and 800 μg/mL vanadate (brown circles). ROS production in non-treated (blue solid diamond) and cells treated with H_2_O_2_ (brown dash) are presented as negative and positive controls respectively. The error bars represent the standard deviation between 3 trials.

The relative levels of ROS generated from selenite and tellurite and a series of other oxyanions (selenate, arsenite, chromate, and vanadate) were evaluated (Figure 2). Regardless of its high MIC, selenite exposure results in the highest ROS production exceeding that of the control hydrogen peroxide. Other oxyanions were added below their MIC levels. Arsenite and selenate provided similar ROS output even though the selenate was 32-fold more concentrated. With arsenite used at one quarter the concentration resulted in a ROS response similar to that observed for 1 μg/mL ${\text{TeO}}_{3}^{2-}$. Neither 13 μg/mL chromate, nor 800 μg/mL vanadate were able to trigger a detectable ROS response in the cells under the conditions used.

**Figure 3:**
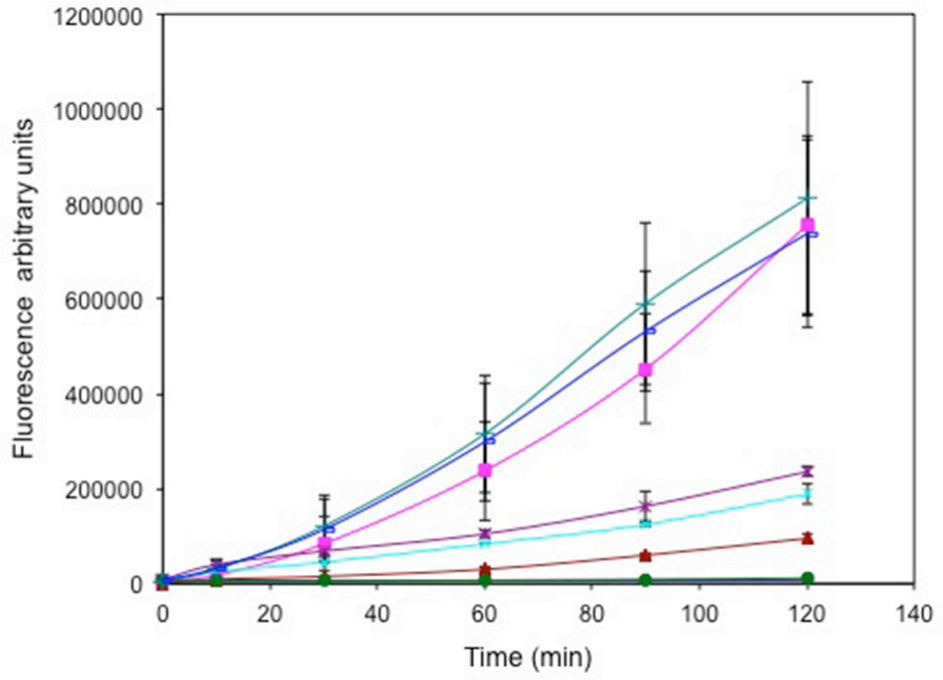
**Time course of reactive oxygen species (ROS) production**. Exponentially growing cells were washed, suspended in saline in the presence of the ROS sensitive probe carboxy-DCFH-DA and exposed to metals. 800 μg/mL selenite (green triangle), 25 μg/mL tellurite/800 selenite (blue line), 1 μg/mL tellurite (brown ^*^), 0.25 μg/mL tellurite (cyan X), 0.125 μg/mL tellurite (brown triangle), 25 μg/mL tellurite (solid green circle). ROS production in non-treated (blue solid diamond) and cells treated with H_2_O_2_ (pink square) are presented as negative and positive controls respectively. The error bars represent the standard deviation between 3 trials.

Cells treated with selenite (±tellurite, Figure 3) exhibited high fluorescence (725,000 and 800,000 a.u. respectively), comparable to cells treated with 1% hydrogen peroxide (725,000 a.u.). Tellurite treated cells exhibited a ${\text{TeO}}_{3}^{2-}$ concentration dependent increased fluorescence reflecting increased ROS production with increased ${\text{TeO}}_{3}^{2-}$ challenge. However, even at the highest oxyanion concentration 1 μg/mL, the fluorescence was only ~ ¼ of the fluorescence seen for the selenite treated cells. Cells treated with 25 μg/mL ${\text{TeO}}_{3}^{2-}$ (well above the MIC) without selenite only showed a background level of fluorescence indicating that at this concentration the cells are killed and hence are not able to mount a ROS response. For the curve of addition of selenite at a level that increased the MIC of tellurite there was not much difference in ROS levels suggesting that ROS production is dominated by selenite.

### Effect of oxyanions on superoxide dismutase (SOD) activity

**Figure 4:**
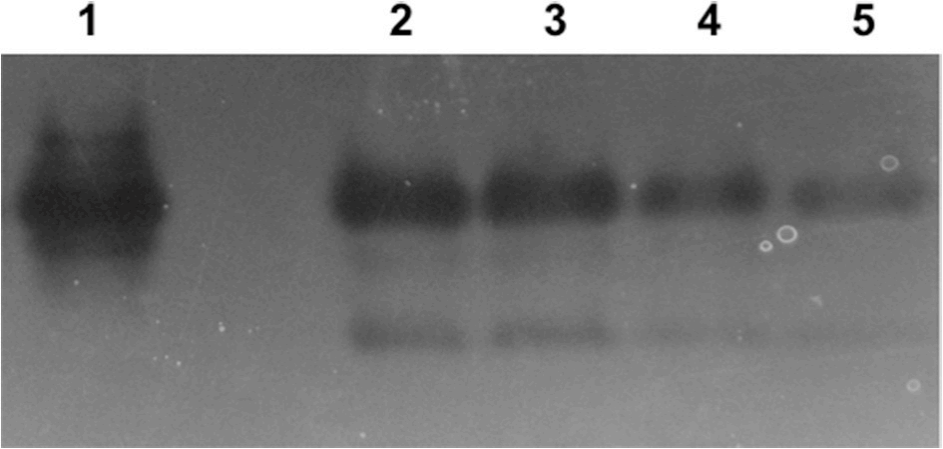
**Superoxide dismutase (SOD) activity in native-PAGE**. Lanes: 1. *E. coli* Mn SOD standard, 2. 800 μg/mL SeO_3_ + 1 μg/mL TeO_3_, 3. 800 μg/mL SeO_3_, 4. 1 μg/mL TeO_3_, 5. control (untreated cells). Lanes 2 through 5 loaded with 50 mg protein from soluble cell fraction.

Superoxide dismutase activity was evaluated using a zymogram in order to determine if selenite or tellurite inhibit or stimulate induction of this enzyme (Figure 4). Tellurite shows only an approximate doubling of the expression of Mn SOD (as evaluated by band intensities lanes 5, untreated control, to lane 4). Selenite and selenite + tellurite were essentially equivalent leading to strong induction and activity of SOD. These results suggest that the selenite protective effect may partially be due to a differential dismutase enzyme accumulation/activity.

### Cell viability kill curves subsequent to metal exposure

**Figure 5:**
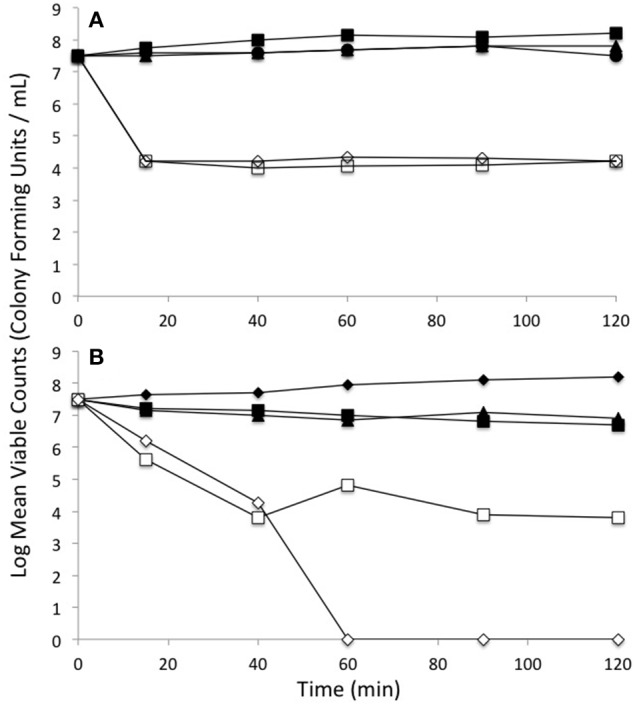
**Loss of viability upon exposure to selenite and tellurite combinations**. **(A)** Wild type *E. coli*: Untreated (solid circle), 1 μg/mL tellurite (open dimond), 800 μg/mL selenite (solid square), 0.25 μg/mL tellurite (open square), 1 μg/ml tellurite with 800 μg/ml selenite (solid triangle). **(B)** Comparison of selenite protection of tellurite toxicity between WT and a strain absent in glutathione production Δ*gshA*: WT strain no exposure (solid diamond), WT strain with 800 μg/ml tellurite and 800 μg/ml selenite (open square), WT strain with 1600 μg/ml tellurite and 800 μg/ml selenite (open triangle), Δ*gshA* strain with 800 μg/ml tellurite and 800 μg/ml selenite (solid triangle), Δ*gshA* strain with 1600 μg/ml tellurite and 800 μg/ml selenite (solid square). The standard deviations between experiments have magnitudes within the size of the plotting symbols.

In order to evaluate the cell killing subsequent to metal exposure, the viability of log phase cells were examined for 2 h post metal addition by colony count plating. The shapes of these kill curves are different between the different conditions and thus are qualitatively compared. Although, the viability of cells treated only with tellurite dropped off rapidly at even 0.25 μg/ml, co-exposure with selenite did not show any loss of viability even with 1 μg/ml tellurite (Figure 5A). This experiment was extended using different ratios of tellurite to selenite. High loads of selenite still provided protection of viability to extremely high loads of tellurite (800 μg/ml) (Figure 5B). The experiment was repeated using the Δ*gshA* strain. In this strain there would be no glutathione as a target of either tellurite or selenite mediated Painter style reactions (2GSH + ${\text{Se0}}_{3}^{2-}$ –> GS-Se-SG) (Painter, 1941). We observe only minor loss of viability of tellurite-exposed cells, co -exposed with 800 μg/mL selenite, even at tellurite concentrations as extremely high as 1600-fold over the MIC.

We note that in the Δ*gshA* and Δ*gor* strains grown in the presence of tellurite had markedly less blackening of the media from Te nanoparticle production (not shown), suggesting that in these strains reduction of tellurite was affected. A similar, though less dramatic, difference in the reduction to red elemental selenium was seen for the selenite treated cells.

## Discussion

Typically bacteria can reduce the toxicity of metals by decreasing their uptake, enhancing their efflux, by sequestration, metabolic by-pass, or through conversion (e.g., reduction/oxidation) to less toxic forms (Trutko et al., 2000; Lemire et al., 2013). For tellurite, reduction to elemental tellurium is evidenced by blackening of cells but the exact mechanism of tellurite reduction remains debated and is likely very different between bacterial species (Zannoni et al., 2008; Borghese et al., 2014; Zonaro et al., 2015). Some studies have proposed that tellurite reduction at the expense of glutathione and other thiols results in blocks in various biosynthetic processes and leads to cell death. Tellurite reduced inside the cell by un-related enzymatic activities, cytosolic glutathione and/or other reduced thiols generates metallic tellurium (Te^0^) and superoxide leading many to propose that oxidative stress in response to metal exposure is one of the major causes of tellurite toxicity to cells (Zannoni et al., 2008; Chasteen et al., 2009). Reduction of selenite also involves reactions with sulfhydryl groups of thiol containing molecules such as glutathione (GSH) resulting in production of GS-Se-SG, GS-she, and HSe^−^ and finally elemental Se^0^ (Turner et al., 1998; Bebien et al., 2002). These reactions can produce damaging ROS molecules and expression of known oxidative stress defense mechanisms in bacterial cells has been shown to contribute to selenite resistance. A cross-protective effect of co-exposure to selenium and mercury has been documented in eukaryotes and has also been exhibited in the soil microbe *Pseudomonas fluorescens* (Belzile et al., 2006), and it is this possibility that we are exploring in this study for *E. coli.*

Some studies have described reductases exhibiting tellurite reducing ability (Moscoso et al., 1998) including nitrate reductases (Avazéri et al., 1997). In *R. sphaeroides* nitrate reductase reduction of tellurite has shown a low catalytic level and resistance could not be associated with the reduction (Sabaty et al., 2001). The nitrate reductases do not appear to be a factor in the current work, as the addition of nitrite or nitrate did not provide any change in MIC of tellurite toxicity and would not have been expected as the experiments here were performed aerobically. In *E. coli* 6-phosphoguconate dehydrogenase (Sandoval et al., 2015) and NDH-II dehydrogenase (Díaz-Vásquez et al., 2014, 2015) appear to have tellurite reduction activity. In these studies, this reduction leads to superoxide production that affects aerobic electron transport chains leading to a move into an anaerobic respiratory state (Molina-Quiroz et al., 2014). Earlier work suggests that Glucose-6-phosphate dehydrogenase plays a protective role in the tellurite mediated oxidative stress (Sandoval et al., 2011). Overall the tellurite mediated stress on *E. coli* also affects the glycolytic pathway, changing the accumulation of various metabolic intermediates (Reinoso et al., 2012; Valdivia-González et al., 2012).

Numerous ‘omic studies have been used to examine bacterial responses to metal toxicity (Bebien et al., 2002; Wang and Crowley, 2005; Brown et al., 2006; Chourey et al., 2008; Booth et al., 2011) and have shown that simultaneous induction of several stress response systems occurs. In *E. coli*, numerous enzymes with antioxidant properties are induced by selenite or selenate treatment including SodA and SodB (involved in degradation of the superoxide anion) and TrxA/TrxB (involved in protection against H_2_O_2_) (Bebien et al., 2002). Examination of chromate stress in *Shewanella oneidensis* revealed a down-regulation of energy metabolism (electron transport components) and a similar up-regulation of functions associated with oxidative stress protection, protein stress protection and DNA repair. An increase in sulfur acquisition and assimilation elements at both the transcriptomic and proteomic level have also been observed (Brown et al., 2006). In *Lactococcus lactis* it has been demonstrated that tellurite induces different effects in two different Trx paralogs. A TrxA mutant exhibited decreased growth rate immediately following tellurite exposure whereas a TrxD mutant showed tellurite induced growth defects on a more long-term basis, indicating a potential role in detoxification (Björnberg et al., 2014; Efler et al., 2015). Here we see that selenite can protect the tellurite toxicity in a Δ*trxA* strain from a MIC of 1 μg/mL to a MIC of 128 μg/ml upon co-exposure with 800 μg/mL selenite. This suggests that the presence of this Thioredoxin may be behaving similar to that of glutathione, synergistically facilitating the toxicity of tellurite.

The structural similarity between chromate, selenite and tellurite and the biologically important anions ${\text{SO}}_{4}^{2-}$ and ${\text{PO}}_{4}^{3-}$ suggests that transport of ${\text{SeO}}_{3}^{2-}$ and ${\text{TeO}}_{3}^{2-}$ across the cell membrane likely occurs via the sulfate and phosphate transport systems. However, uptake has also been shown to be mediated by carboxylate transporters (Borghese et al., 2008). A transport blocking effect cannot however be the sole mechanism of selenium-protection, as all strains would then exhibit an equivalent level of protection.

An observation in this work was that ROS production does not correlate with MIC levels for different metals and increased ROS production does not necessarily correlate with increased metal toxicity. The addition of selenite resulted in higher ROS production (similar to that of hydrogen peroxide addition) than tellurite, although the latter is considerably more toxic to cells. This calls into question the belief that a large part of tellurite toxicity is attributable to its inducing an oxidative stress in cells. Cell death seems to occur at low concentrations of tellurite thus not providing enough oxyanion to catalyze high concentrations of ROS. The possibility exists that the low tellurite concentration of cell killing does not sufficiently trigger stress response mechanisms in the cell, limiting the cell's ability to mount an adaptive response to the metal. However, in a metabolomics investigation of a strain of *Pseudomonas pseudoalcaligenes KF707*, tellurite resistance correlated with the induction of oxidative stress response, resistance to membrane perturbation and reconfiguration of the cellular metabolism, particularly increased levels of glutathione (Tremaroli et al., 2009).

The protective effect of selenite may partially be attributed to its ability to trigger a greater oxidative stress and hence a stronger adaptive response but this is not the sole mechanism, as a similar protective effect would exist with other oxyanion combinations. Potential protection mechanisms include but are not limited to: triggering a stronger acute adaptive response (e.g., such as the stronger SOD response observed in this work) which is then protective against tellurite, preferential binding of selenite to tellurite target sites (these selenite products being less toxic), and alternate metabolic flux.

It is clear from this work that ROS associated cell damage is only part of the metal toxicity story. The ability of oxyanions to react with sulph-hydryl groups in protein cysteines and methionines as well as Fe-S clusters can result in disruption of function of a variety of proteins including components of the electron transport chain. The contribution of such interactions to tellurite toxicity is indicated by the increased sensitivity of cysteine desulfurase, *iscS*, mutants (Tantaleán et al., 2003) and other cysteine metabolism genes (e.g., *cysK*) in *E. coli* and other bacteria (O'Gara et al., 1997; Fuentes et al., 2007).

A number of roles for CydDC have been proposed and refuted including cysteine transport and haem transport (Cook and Poole, 2000). Strains mutated in *cydD* lack periplasmic cytochromes *c* and do not assemble cytochrome *b*_562_. CydD is not however needed for synthesis of haem D or assembly of CydA/CydB (encoding quinol oxidase cytochrome *bd*) (Cook and Poole, 2000). CydD has been shown to function as a GSH transporter and to be important for assembly of cytochrome *bd* quinol oxidase (Pittman et al., 2005). This discovery indicates that GSH plays an important role in redox homeostasis in the periplasm and does not act alone in the cytoplasm. Cytochrome *bd* has been shown to be induced when *E. coli* is grown under unfavorable growth conditions (Kato et al., 1996) and CydD mutants fail to synthesize periplasmic *c-*type cytochromes which are needed under anaerobic conditions or with alternate electron acceptors such as nitrite, as well as cytochrome *bd* oxidase (Cook et al., 1997). The apparent lack of interference with tellurite reduction in a CydD mutant (indicated by blackening of growth media) disproves previous suggestions that cytochrome *b* (or *d)* is involved in tellurite reduction. Yet we see increased tellurite toxicity protection by selenite in a Δ*cydD* strain.

There have been studies into the role of the Dsb system proteins in cytochrome *c* maturation, particularly through the CcmG/H pathways (Stirnimann et al., 2006). Pittman et al. (2005) in discovering that CydDC transports GSH to the periplasm, speculated that it mediated its activity by compensating for DsbD. The impaired ability of Δ*gshA* and Δ*gor* mutants to reduce tellurite supports a more direct effect of GSH on cytochrome proteins and reduction as has been previously suggested (Borsetti et al., 2007). Furthermore, the highly enhanced tellurite resistance with selenium protection in the Δ*gshA* mutant suggests that the presence of GSH actually enhances tellurite toxicity and is similarly seen in Δ*cydD* (Table 1). This is in agreement with the earlier work that suggested that the presence of *gshA* decreases survivability of *E. coli* by tellurite where as no other thio-disulphide metabolism gene showed a similar affect (Harrison et al., 2009).

The apparent slightly greater selenite effect on RSH reduction may largely be related to the 800-fold higher addition of this metal relative to tellurite. The selenite driven reduction in RSH supports the observation of a greater oxidative stress with this metal. At the sensitivity tested, no additive effect of loss in RSH was observed in the double metal treatments. These results suggest that the increased toxicity of tellurite is not directly related to a greater reduction in the reduced thiol pool although it may be the nature of the products formed and not the concentration that results in toxicity.

This work presents the observation of a protective phenomenon rather than a synergistic destructive effect of the presence of the two metalloids selenite and tellurite. In addition this work shows that increased resistance observed by mutants of the various thiol redox pathway components suggests that metal reactions with thiol components such as GSH and associated oxygen radical production can actually enhance toxicity of certain metals. Alternatively, increased disulphide and oxidative stress associated with the disruption of the thiol redox buffering pathways may prime cell adaptive responses improving microbial survivability to metals.

## Author contributions

HV was a PDF on the project leading the experiments, SW did technical work to support HV and followed up with key experimental repeats. BH was an undergraduate summer research student that did a lot of exploratory experiments. RT was the professor leading the project that advised and rethought experiments. HV and RT wrote the manuscript.

### Conflict of interest statement

The authors declare that the research was conducted in the absence of any commercial or financial relationships that could be construed as a potential conflict of interest.
